# 18F-FDG /18F-Choline Dual-Tracer PET Behavior and Tumor Differentiation in HepatoCellular Carcinoma. A Systematic Review

**DOI:** 10.3389/fmed.2022.924824

**Published:** 2022-07-07

**Authors:** Jérôme Ghidaglia, Nicolas Golse, Alina Pascale, Mylène Sebagh, Florent L. Besson

**Affiliations:** ^1^Department of Biophysics and Nuclear Medicine-Molecular Imaging, Hôpitaux Universitaires Paris-Saclay, Assistance Publique-Hôpitaux de Paris, Le Kremlin-Bicêtre, France; ^2^Centre Hépato Biliaire, Hôpitaux Universitaires Paris-Saclay, Assistance Publique-Hôpitaux de Paris, Villejuif, France; ^3^Université Paris-Saclay, INSERM, Physiopathogénèse et Traitement des Maladies du Foie, UMR-S 1193, Gif-sur-Yvette, France; ^4^Department of Pathology, Hôpitaux Universitaires Paris-Saclay, Assistance Publique-Hôpitaux de Paris, Le Kremlin-Bicêtre, France; ^5^Université Paris-Saclay, School of Medicine, Le Kremlin-Bicêtre, France; ^6^Université Paris-Saclay, CEA, CNRS, Inserm, BioMaps, Orsay, France

**Keywords:** HCC, PET, FDG, choline, molecular imaging

## Abstract

**Background:**

Post-operative recurrence remains the strongest prognostic factor of resected hepatocellular carcinoma (HCC), making the accurate selection of patients with curable HCC a crucial issue. PET imaging combining both 18F-FDG and fatty acid synthase (FAS) radiotracers—such as Choline—has shown its interest for the initial staging and therapeutic management of patients with HCC, but its use is still not consensual. Importantly, the very first dual-tracer PET studies suggested 18F-FDG/FAS PET behavior be linked to the degree of differentiation of HCC, a major predictive factor of post-operative recurrence. Although this key molecular imaging concept may impact how dual-tracer PET will be used in early-stage HCC, its level of evidence remains largely unexplored. In this study, we conducted a systematic review of the available evidence-based data to clarify the relevance of dual 18F-FDG/18F-Choline PET in characterizing the degree of differentiation of HCC tumors.

**Methods:**

A systematic search of the PubMed/Medline and Embase databases was performed up to November 2021. A systematic review of the dual-tracer 18F-FDG/18F-Choline PET behavior of histology-proven HCC according to their degree of differentiation was conducted. The overall quality of the included studies was critically assessed based on the STROBE guidelines. Information on study date, design, patient cohort characteristics, grade of differentiation of HCC tumors, and the dual-tracer PET behavior per HCC was independently extracted and summarized.

**Results:**

From 440 records initially available, 6 full-text articles (99 histology-proven HCC) provided dual-tracer 18F-FDG/18F-Choline PET behavior per HCC tumor grade were included in the systematic review. Based on our analysis, 43/99 HCCs were reported to be well-differentiated, and 56/99 HCCs were reported to be less-differentiated tumors. In the well-differentiated subgroup, more than half were exclusively positive for 18F-Choline (51%), whereas 39% were positive for both 18F-FDG and 18F-Choline. In the less-differentiated subgroup, 37% of HCC patients were positive exclusively for FDG, 36% were positive for both 18F-FDG and 18F-Choline, and 25% were positive exclusively for 18F-Choline.

**Conclusion:**

The 18F-FDG/18F-Choline dual-tracer PET behavior of uptake shows high overlap between well- and less differentiated HCC, making the characterization of tumors challenging based on such PET combination alone. Given our growing knowledge of the molecular complexity of HCC, further studies are necessary to refine our understanding of radiotracers’ behavior in this field and improve the usefulness of PET imaging in the clinical decision process of HCC.

## Introduction

Hepatocellular carcinoma (HCC), the most common primary liver cancer and the third leading cause of cancer death worldwide, represents a major health challenge (1). Chronic liver disease—cirrhosis—which can be related to alcohol, NASH, HBV or HCV infection, or less frequently primary biliary cholangitis, hemochromatosis, or α1-antitrypsin deficiency, is the strongest risk factor of HCC development, concerning more than 90% of the cases. The treatment strategy is currently mainly driven by the Barcelona Clinic Liver Cancer staging system (BCLC), a five-stage classification scale integrating the characteristics of the primary tumor (size, number of nodules), the disease extent (portal invasion, N+ and M+ status), the liver function (Child-Pugh), and the performance status (ECOG). Very early and early-stage HCC patients may benefit from various curative options (resection, ablation or liver transplantation), whereas more advanced stages are candidate to chemoembolization, systemic therapies including immunotherapy, or best supportive care. In early-stage HCC, post-operative recurrence remains the strongest survival prognostic factor (2) making the accurate selection of patients with curable HCC a crucial issue. Tumor differentiation is a major predictive factor of post-operative recurrence in HCC (3, 4). However, the histological analysis of tumor differentiation, which remains the gold standard, is currently carried out only in atypical cases. Conventional imaging is essential for the management of HCC patients (5–8), but its limited value in such atypical cases and the need for non-invasive biomarkers of tumor differentiation have progressively motivated the use of PET imaging in this field (9), while this functional imaging is still not consensually recommended. Because 18F-fluorodeoxyglose (18F-FDG) shows mitigated performance to detect HCC (10, 11) but excellent specificity for HCC metastases (9), PET radiotracers of fatty acid synthase (FAS) have been proposed as complements, such radiolabeled choline, the most widely used FAS-targeted radiotracer in clinical practice (12, 13). Although 18F-FDG and FAS PET radiotracers have shown their complementarity for the initial staging and treatment management of HCC patients (14–17) their combined use is still not consensual, making dedicated recommendations challenging (18).

The very first dual 18F-FDG/18F-Choline PET studies suggested 18F-FDG/FAS PET behavior be linked to the degree of differentiation of HCC tumors (19, 20). Although this key concept could impact the rational for using dual-tracer PET in HCC, its level of evidence remains largely unexplored. In this study, we conducted a systematic review of the available evidence-based data, to clarify whether ^18^F-FDG/^18^F-Choline dual-tracer PET behavior is a relevant imaging biomarker of tumor differentiation in HCC.

## Methods

This methodological study was conducted according to the PRISMA 2020 statement for systematic review reports (21).

### Search Strategy

Two authors (JG and FLB) independently performed a comprehensive search of PubMed/Medline and Embase databases to find studies using 18F-FDG and 18F-Choline dual PET tracers for HCC purposes. The search strategy combined the following keywords: “HCC + PET” or “HCC + FDG” AND “HCC + Choline.” No starting date was used, and the search procedure was updated until 10 November 2021. Moreover, references of the retrieved articles were also screened for additional studies. The inclusion criteria were as follows: (i) articles exclusively in English; (ii) the combined use of 18F-FDG and 18F/11C-Choline dual PET tracers for each HCC tumor, in order to assess the dual-tracer PET behavior without a priori; (iii) histology-confirmed HCC diagnosis; and (iv) available description of HCC differentiation for each patient. All the articles not fulfilling the inclusion criteria mentioned above, together with review articles, editorials, letters, comments, or case reports, were excluded from the analysis. For each eligible study, the following information was independently extracted: study date; design; patient cohort characteristics, including sample size, number of patients with HCC, and number of HCC lesions per patient confirmed by histology (either by surgery or biopsy); delay time between the 18F-FDG and 18F-Choline PET acquisitions; histological differentiation of HCC; and dual PET radiotracer behavior of HCC patients per histological subtype. For all included studies, the same predefined definition of PET positivity was considered: any focal uptake superior to the locoregional background was considered positive, whereas iso or hypometabolic lesions were considered negative for the radiotracer of interest.

### Quality Assessment of the Studies

The overall quality of each included study was critically assessed by two authors (JG and FLB) based on the “STROBE guidelines” (22). Because the dual PET tracer behavior according to the level of differentiation of HCC was never considered the primary outcome, a general standardized checklist of 22 items covering the overall quality statements of non-interventional studies (22) was independently applied by the two readers as follows: each item was quoted “yes” if present, “no” if absent, or “unclear” if the statement was equivocal. All disagreements between the two readers were resolved by consensus.

### Ethics and Data

The need for ethical approval was waived due to the nature of the study (review article).

## Results

### Literature Search

**FIGURE 1:**
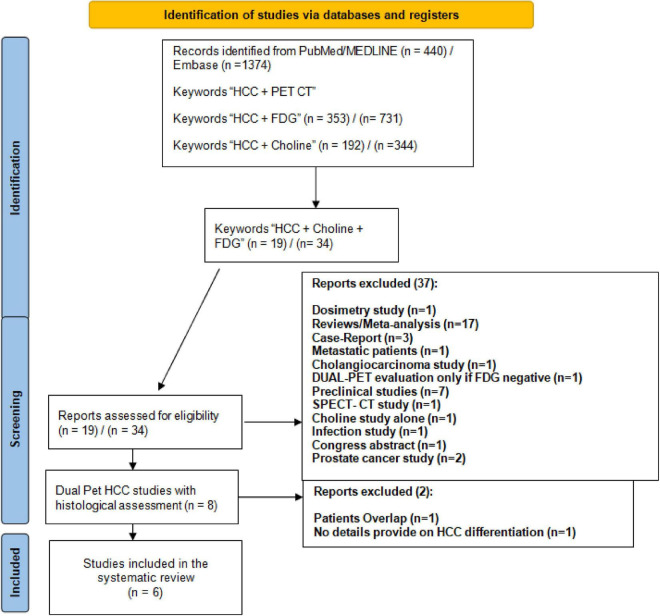
PRISMA flowchart of the systematic review.

The PRISMA flow diagram of the literature search is provided in Figure 1. The comprehensive literature search from PubMed/MEDLINE and Embase identified the following records: 440 records using the “HCC + PET CT” keywords; 353 records using the “HCC + FDG” keywords; 192 records using the “HCC + Choline” keywords; and 19 records using the “HCC + FDG + Choline” keywords. Among the 19 articles, 12 were discarded due to study type (reviews *n* = 4), analysis of metastatic disease (*n* = 1), cholangiocarcinoma study (*n* = 1), use of Choline only in 18F-FDG-negative patients (*n* = 1), and preclinical studies (*n* = 2). At the end of the screening process, 7 full-text articles were retrieved. Among them, one article was discarded because no explicit HCC differentiation status per patient was provided (23), and two studies presented patient data overlap (20, 24). Of the two studies with patient data overlap, we discarded the second study (24). Finally, 6 full-text articles over the last 15 years (2006–2021) were included in the systematic review (14, 16, 19, 20, 25, 26).

### Methodological Quality of the Included Studies

**FIGURE 2:**
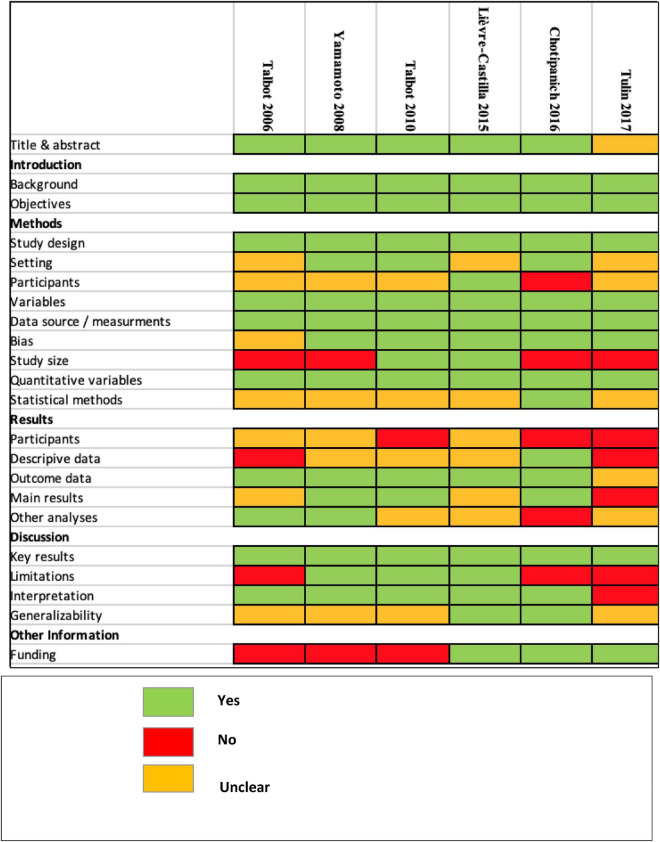
STROBE quality assessment.

The overall quality assessment of the 6 included studies is shown in Figure 2. The studies were of limited quality with 12 items (55%) considered present (range = 9–16), 3 items (14%) considered absent (range = 0–6) and 6 items (27%) considered unclear after a double blinded reading (range = 5–11). In particular, only two studies provided a majority of unequivocal item statements for the results section (19, 26), and one study provided unclear or no information for the majority of the STROBE statements of the discussion section (25). Considering these statements, the level of evidence for the dedicated use of dual 18F-FDG/18F-Choline PET tracer for the characterization of HCC differentiation was considered a level 4–5 (grade C-D of recommendation) according to the Oxford Centre for Evidence-Based Medicine.

### Qualitative Analysis (Systematic Review)

The main characteristics of the 6 included studies are provided in Table 1. The primary outcomes were as follows: the detection rate of 18F-FDG and 18F-Choline PET tracers for HCC tumors (16, 19); the diagnostic performance of 18F-FDG and 18F-Choline PET tracers for detecting and staging HCC in patients with chronic liver disease (14, 20); and the assessment of dual 18F-FDG and 18F-Choline PET with perfusion CT behaviors of HCC tumors (25). The dual radiotracer PET data of 99 biopsy-proven HCC patients were reanalyzed. The study samples were mainly limited with only two prospective studies reaching more than 20 histologically proven HCC samples (14, 20). Talbot et al. (20) included a case mix of 34 HCC patients (27 biopsy proven), 2 hepato-cholangiocarcinoma patients, 10 patients with other malignancies, and 8 patients with benign conditions. Castilla-Lièvre et al. (14) included 38 HCC patients (22 biopsy proven) with 4 cholangiocarcinoma patients and one adenoma patient. The other 4 studies exclusively focused on HCC patients. Among the pooled 99 HCC tumors, 43 were considered well-differentiated tumors, and 56 were considered moderate to poorly differentiated tumors. The classification used for tumor differentiation (Edmonson Steiner or WHO) was mentioned for only 57% of the HCC tumors as follows: biopsy for 14 HCC tumors (14, 16) surgery for 37 HCC tumors (14, 19), and a case mix of biopsy/surgery for the rest of the dataset without any information concerning the ratio. Additionally, 50 HCC patients were explicitly qualified as having unifocal HCC tumors (14, 16, 19), and 25 HCC patients were explicitly qualified as having multifocal HCC tumors (14, 16, 19, 25). In all studies, PET positivity was defined visually as non-physiological focal liver radiotracer uptake in four studies (14, 16, 19, 25), whereas one study also proposed a “photopenic” pattern for Choline PET positivity (20).

**TABLE 1:** Characteristics of the included studies with dual PET tracer.

References	Country	Design	HCC patients	Histology proven HCC	Delay time between the two PETs
Talbot et al. (16)	France	prospective	9	7	1 Week
Yamamoto et al. (19)	Japan	retrospective	12	16	2 Weeks
Talbot et al. (20)	France	prospective	34	27	1–4 Weeks
Castilla-Lièvre et al. (14)	France	prospective	38	22	0–2.5 Weeks
Chotipanich et al. (26)	Thailand	prospective	12	9	Unknown
Tulin et al. (25)	Russia	retrospective	18	18	Unknown
Total			111	99	

*Patients who underwent dual PET tracers exclusively. Histology was confirmed by either surgery or tumor biopsy.*

The 18F-FDG and 18F-Choline PET tumor behaviors according to the differentiation of the tumors are shown in Table 2. In the well-differentiated subgroup, 51% were exclusively positive for 18F-Choline, 39% were positive for both 18F-FDG and 18F-Choline, and only 5% were exclusively positive for FDG. Two patients were negative for the two PET radiotracers (20, 26). In the poorly differentiated subgroup, 37% of HCC patients were exclusively positive for 18F-FDG, 36% were positive for both 18F-FDG and 18F-Choline, and 25% were exclusively positive for Choline. One patient was considered negative for both radiotracers (20).

**TABLE 2:** Dual PET radiotracer behaviors function of HCC level of differentiation.

References	HCC	Grade of tumor differentiation							
		
		Well differentiated				Moderate-poorly differentiated			
			
		Ch+/FDG−	Ch−/FDG+	Ch+/FDG+	Ch−/FDG−	Ch+/FDG−	Ch−/FDG+	Ch+/FDG+	Ch−/FDG−
Talbot et al. (16)	7	3/4	0/4	1/4	0/4	0/3	0/3	3/3	0/3
Yamamoto et al. (19)	16	0/0	0/0	0/0	0/0	8/16	6/16	2/16	0/16
Talbot* et al. (20)	27	6/11	1/11	4/11	0/11	2/16	4/16	9/16	1/16
Castilla-Lièvre et al. (14)	22	11/16	1/16	3/16	1/16	4/6	2/6	0/6	0/6
Chotipanich et al. (26)	9	2/6	0/6	3/6	1/6	0/3	1/3	2/3	0/3
Tulin^#^ et al. (25)	18	0/6	0/6	6/6	0/6	0/12	8/12	4/12	0/12
Total	99	22/43 (51%)	2/43 (5%)	17/43 (39%)	2/43 (5%)	14/56 (25%)	21/56 (37%)	20/56 (36%)	1/56 (2%)

*Because its biological significance is currently unknown, photopenic lesions on Choline were not considered positive here.

*^#^In this study, the tumor to liver ratio was assessed based on the quantitative data provided. A ratio > 1 was considered positive.*

Five studies also performed SUV-based semiquantitative analyses (14, 16, 19, 25, 26) with discrepant results. In one study, no significant difference was observed between the 18F-FDG or 18F-Choline signal-to-noise ratio irrespective of the HCC level of differentiation (16). Yamamoto et al. found no significant difference in radiotracer uptake between the histological subgroups (19). Tulin et al. reported a significant difference in radiotracer uptake according to the liver areas (well-differentiated HCC, poorly differentiated HCC, or normal parenchyma) (25). Chotipanich et al. found a significant difference between well- and poorly differentiated HCC only for ^18^F-FDG (26). The last study did not perform any comparative analyses (14).

## Discussion

Based on our analysis of the literature data, well-differentiated HCC patients were positive for ^18^F-FDG in approximately 44% of the reported cases and positive for 18F-Choline in 90% of the reported cases. For the less-differentiated HCC tumors (moderate to poorly differentiated HCC), 73% of reported HCC cases were 18F-FDG-positive, and 61% of reported HCC cases were ^18^F-Choline-positive. Among the 56 less differentiated tumors considered in the present study, 16 were explicitly mentioned as poorly differentiated HCC, of which 100% were 18F-FDG-positive and 31% were 18F-Choline-positive. However, the 8 poorly differentiated tumors all considered 18F-FDG-positive and 18F-Choline-negative in one study had no information regarding the motivation of their inclusion criteria (25). Focusing on the 8 remaining poorly differentiated HCC patients (16, 19, 26), 100% were reported to be positive for 18F-FDG, of whom 63% were also positive for 18F-Choline. Moreover, more than one-third of patients showed dual PET tracer positivity regardless of the degree of HCC differentiation.

A trend toward an inverse gradient of dual-PET HCC tumor behavior has been previously suggested (16, 20), but our systematic review showed that a strong mirrored dual-PET tumor behavior according to HCC differentiation should be considered with caution. First, the lack of standardized histological grading of HCC remains a major issue. Although Edmonson Steiner and WHO classifications share similarities (4-level grades and structural/cellular features), the respective definitions for well- and less differentiated grades are not fully concordant with a related impact on subgroup outcomes (27). In our systematic review, only two of the six included studies explicitly mentioned either Edmonson Steiner (19) or WHO (14) classifications (39% of the included HCC). As 61% of the biopsy-proven reported HCC cases were potentially a blinded case mix of ES/WHO definitions, any generalizability of 18F-FDG/18F-Choline dual PET tracer behavior according to HCC grading would be ambiguous. Second, a significant association between glucose metabolism assessed by FDG PET and microvascular invasion (MVI), another strong prognostic factor in HCC (28, 29), has been widely reported (23, 30–32). Surprisingly, Kornberg et al. reported that 14 of the 16 HCC patients who were positive for 18F-FDG PET had MVI (87.5%), of whom only 1/3 were poorly differentiated (5 patients) (30). Although an extensive review by Gouw et al. showed that high-grade tumors but also tumor size and number of nodules to be predictive of MVI (33), MVI has also been reported in 29% of HCCs with a size ranging from 2 to 5 cm (34). Sabaté-Llobera et al. reported that the ratios of well- to less differentiated tumors ranged from 1.3 in the 18F-FDG-positive group to 5.7 in the 18F-FDG-negative group (31). However, nearly 50% of 18F-FDG-positive cases in the present study were well-differentiated HCC. Consequently, ^18^F-FDG PET positivity may reflect both MVI and tumor differentiation, which are two prognostic factors that are not highly interlinked. Third, studies by Okazumi, Torizuka, and Trojan (35–37) have suggested that a loss of FDG 6-phosphatase activity in undifferentiated tumor cells explains the higher 18F-FDG avidity of poorly differentiated HCC. Importantly, the studies by Okazumi and Torizuka were mainly dynamic PET studies (35, 36). The tissue behavior of advanced PET kinetic parameters, especially k3 or k4 microparameters, cannot be directly extrapolated to static PET metrics, such as SUV, a surrogate of the glucose retention index in cells. Trojan et al. reported that the 18F-FDG uptake (SUV) was more efficient not only in poorly differentiated tumors but also in large tumors and elevated AFP (37). In particular, the vast majority of the reported FDG-positive tumors also showed multiple nodules, all being predictive factors of MVI (38).

Evidence-based analysis of the literature over the past 20 years suggests complex interlinks between tumor grade and MVI, and the related FDG PET behaviors in HCC patients. In contrast, 18F-Choline appears not as informative as 18F-FDG to characterize tumor aggressiveness in HCC patients. In 29 HCC patients, Mulé et al. showed a higher 18F-FDG uptake for MVI-positive HCC cases compared to MVI-negative HCC cases (SUVr 2.65 vs. 1, *p* = 0.003) without any significant difference in 18F-Choline uptake (39). This lack of prognostic significance of 18F-Choline was also reported by Castilla-Lièvre et al. (14). Notably, a combined photopenic 18F-Choline with a positive 18F-FDG-PET pattern has been suggested to be a pejorative prognostic factor of HCC recurrence (24). In light of this methodological review, the question of the leading prognostic value of 18F-FDG PET arises. The biological significance of glucose- and Choline-based PET tracer behaviors in HCC patients remains poorly understood.

Recently, a better understanding of biological pathways of HCC tumors has led to the emergence of a new molecular-based classification of HCC, dichotomizing the tumors into proliferation and non-proliferation classes based on their multidimensional molecular pattern (40–45). While the proliferative class is characterized by poorly differentiated tumors, high vascular invasion, and elevated AFP, the non-proliferative class corresponds to well to moderately differentiated tumors, less vascular invasion, and lower level of AFP. In both groups, the characteristics of T-cell infiltrates further define four immune-related subclasses (46). Because the characterization of tumor heterogeneity at the molecular level is emerging in HCC (47, 48), the powerful capabilities of vectorized PET molecular imaging in this field would gain in relevance. Although several 18F-FDG/FAS studies emphasize the clinical usefulness of PET to manage HCC patients (17, 49), future multitracer PET studies are mandatory to better understand the deep biological meaning of multitracer PET behavior in this field.

Our systematic review had several limitations. The limited number of dual 18F-FDG/18F-Choline PET articles with available per patient-based HCC tumor differentiation hampered any quantitative analysis. However, our pooled semiquantitative analysis revealed the high overlap of 18F-FDG/18F-Choline PET behavior between well- and less differentiated HCC. Additionally, we did not include acetate PET studies in this systematic review (11, 50–52). Initially, evaluated in cardiac (53) and urological oncology settings (54, 55), acetate shows a biodistribution quite similar to that of Choline. Although both substrates are fed into fatty acid synthesis, also known as the Kennedy pathway (56–58), Choline and acetate have various other biological functions (59, 60), making them not strictly comparable. Additionally, ^18^F-Choline is currently the most widely used FAS-targeted PET tracer of HCC in clinical practice, which is why we focused on this PET radiotracer in this study.

## Conclusion

The 18F-FDG/18F-Choline dual-tracer PET behavior of uptake shows high overlap between well- and less differentiated HCC, making the characterization of tumors challenging based on such PET combination alone. Given our growing knowledge of the molecular complexity of HCC, further studies are necessary to refine our understanding of radiotracers’ behavior in this field and improve the usefulness of PET imaging in the clinical decision process of HCC.

## Author Contributions

All authors: design, acquisition analysis, revising for intellectual content, final approval, and agreement to be accountable for all aspects of this work (accuracy and integrity of any part of the work).

## Conflict of Interest

The authors declare that the research was conducted in the absence of any commercial or financial relationships that could be construed as a potential conflict of interest.

## Publisher’s Note

All claims expressed in this article are solely those of the authors and do not necessarily represent those of their affiliated organizations, or those of the publisher, the editors and the reviewers. Any product that may be evaluated in this article, or claim that may be made by its manufacturer, is not guaranteed or endorsed by the publisher.

## Abbreviations

PET: Positron emission tomography
HCC: Hepatocellular carcinoma
18F-FDG: 18F-Fluorodeoxyglucose
FAS: Fatty acid synthase
BCLC: Barcelona Clinic Liver Cancer staging system
NASH: Non-Alcoholic SteatoHepatitis
HBV: Hepatitis B Virus
HBC: Hepatitis C Virus
SUV: Standard Uptake Value.
